# Role of Antioxidant Supplementation in Enhancing Chelation Therapy for Lead-Induced Oxidative Stress in Rats

**DOI:** 10.7759/cureus.79699

**Published:** 2025-02-26

**Authors:** Sophiya S Waidande, Mandakini Kshirsagar, Vandana M Thorat, Devkumar D Tiwari

**Affiliations:** 1 Biochemistry, Krishna Institute of Medical Sciences, Karad, IND; 2 Pharmacology and Therapeutics, Krishna Institute of Medical Sciences, Karad, IND; 3 Pharmacology, Krishna Vishwa Vidyapeeth (Deemed to be University), Karad, IND

**Keywords:** antioxidant supplementation, chelation therapy, glutathione, lead toxicity, malondialdehyde, oxidative stress, superoxide dismutase, vitamin c, vitamin e, wistar rats

## Abstract

Background and aim

Lead poisoning is an increasingly critical issue in environmental and public health due to its ability to induce oxidative stress (OS), which contributes to various diseases. While chelation therapy is widely used to lower blood lead levels, it does not fully counteract the OS caused by lead exposure. Recent research suggests that combining chelation therapy with antioxidants may enhance its effectiveness by mitigating oxidative damage. This study evaluates whether supplementing chelation therapy with vitamin C can improve its efficacy in reducing OS in lead-exposed rats.

Material and methods

Forty male Wistar rats were divided into four groups: a control group, a lead-exposed group, a chelation therapy group, and a group that was given both chelation therapy and antioxidants. Serum malondialdehyde (MDA) levels and superoxide dismutase (SOD) activity, catalase activity, and glutathione (GSH) levels were determined to assess the efficiency of the treatment.

Results

The outcomes revealed that chelation therapy lowered some of the OS indicators, but this was much more pronounced when carrying out chelation therapy in combination with antioxidant intake. More particularly, the combination therapy group had an MDA level closer to control and significantly higher SOD and catalase activity, and nearly normal GSH levels.

Conclusion

The results of the present study indicate that antioxidant supplementation might provide synergistic support to chelation therapy for the management of lead-induced OS.

## Introduction

Lead poisoning still prevails in the environment and accounts for population health problems, especially in areas with large industrial facilities. Lead causes OS, which is harmful to human health and can lead to neurotoxicity, nephrotoxicity, and reproductive toxicity, according to Gurer and Ercal (2000). OS is defined as a state in which there is an elevated intracellular level of reactive oxygen species (ROS) along with the reduced capability of the body to neutralize these toxic intermediates or repair the resulting deterioration. Lead exposure triggers the production of excessive ROS, which causes lipid peroxidation, protein oxidation, and DNA damage, disrupting normal cell function and leading to apoptosis [1,2].

The common treatment for lead poisoning is chelation, in which the lead is enclosed with agents such as ethylenediaminetetraacetic acid (EDTA) and then expelled from the body. However, the use of chelation therapy only appears to be incomplete in rehabilitating the OS elicited by lead toxicity. Some papers have pointed out that adding antioxidants may improve chelation therapy outcomes due to reduced OS, thus presenting a multiaxis therapy [3,4].

Chelation therapy for lead poisoning

Chelation therapy continues to be the principal approach to reducing lead loads in a patient’s system. Chelating agents, which include EDTA and dimercaptosuccinic acid (DMSA), help in the chelation of lead ions to be expelled through the urine. However, although chelation therapy does decrease blood lead concentrations, it is inadequate in reversing the OS produced by lead exposure [5]. Studies have concluded that OS markers, such as malondialdehyde (MDA), remain elevated after chelation therapy, indicating persistent oxidative damage [6]. This highlights the need for a more comprehensive treatment approach that addresses both lead detoxification and the oxidative damage it causes.

Recent studies suggest that antioxidant supplementation could improve the outcomes of chelation therapy by reducing OS. Antioxidants, including vitamins C and E, neutralize ROS and support the body's endogenous antioxidant defense system [7]. For instance, vitamin E is known to reduce lipid peroxidation, while vitamin C has been shown to regenerate other antioxidants and directly scavenge free radicals [8]. Co-supplementation of chelation therapy with antioxidants has been proposed as a multi-axis therapy that removes lead and mitigates the oxidative damage caused by lead exposure [9]. This combined approach could offer more effective clinical results than chelation therapy alone, as it addresses lead detoxification and OS.

Several studies have experimented with using natural antioxidants and their combination with chelating agents. For example, when combined with chelators, flavonoid-based compounds have been shown to enhance detoxification and antioxidant capacities [10]. Curcumin, a natural antioxidant, has also been demonstrated to reduce OS and improve treatment outcomes in models of lead poisoning [11]. Moreover, advancements in therapeutic strategies, such as nanoparticles and newer chelating agents, have been offered as promising alternatives to conventional approaches. Nano-curcumin combined with monoisoamyl 2,3-dimercaptosuccinic acid (MiADMSA) has shown therapeutic advantages in reducing OS and lead toxicity [12], and newer chelators, such as N,N'-bis-(2-mercaptoethyl) isophthalamide (NBMI), have exhibited neuroprotective effects in lead-exposed models [13].

However, despite the promising results of these studies, there remains a limited understanding of the combined effects of antioxidants and chelation therapy on lead-induced OS. The interaction between antioxidants and chelating agents and their potential to improve clinical outcomes by addressing oxidative damage requires further exploration. In a rat model of lead poisoning, the current study aims to determine whether or not the addition of vitamin C to chelation therapy will decrease the number of OS markers. These markers include MDA levels, SOD activity, catalase activity, and glutathione (GSH) levels. We hypothesize that the combined therapy will result in more significant decreases in OS when compared to the chelation therapy alone, which will ultimately result in a more effective treatment strategy for lead poisoning.

This work examines the effects of antioxidant supplementation in potentiating chelation therapy's impact in a rat-lead OS model. The working hypothesis of the study is that combination therapy of antioxidants with chelation therapy produces a higher level of significance in decreasing the parameters of OS compared to chelation therapy alone.

## Materials and methods

Experimental animals

Twenty-four adult Wistar rats, weighing between 180-220 g, were used in this study. The rats were housed under standard conditions with a 12-hour light/dark cycle and had free access to food and water. All experimental procedures were conducted following ethical guidelines for animal research and were approved by the Institutional Animal Ethics Committee of Krishna Institute of Medical Sciences (approval number: 2017/7/17).

Lead exposure and treatment protocols

For groups exposed to lead, lead acetate was administered in drinking water for four weeks. After this exposure period, different treatment protocols were followed. The chelation therapy group received EDTA (75 mg/kg body weight) administered intraperitoneally once daily for one week. The combination therapy group underwent the same lead acetate exposure, followed by chelation therapy with EDTA (75 mg/kg body weight) and antioxidant supplementation with Vitamin C (500 mg/kg body weight), administered orally once daily for one week.

Sample collection

At the end of the treatment period, the rats were anesthetized and sacrificed by euthanasia using isoflurane inhalation, as per methods selected based on previous animal experiment studies. Blood samples were collected via cardiac puncture, and tissues were collected for analysis. All facilities required for the procedure were available at Krishna Institute of Medical Sciences, Karad. The blood samples were processed immediately in the biochemistry lab under the proper guidance of experts using semi-automated machinery, and all samples were stored at -80°C for further analysis.

Biochemical assays

The following OS markers were measured in the blood samples. MDA levels were assessed as an indicator of lipid peroxidation using the thiobarbituric acid reactive substances method, with results expressed as nmol/mg protein [14]. SOD activity was measured to evaluate the enzyme’s ability to neutralize superoxide radicals using the nitroblue tetrazolium reduction method and expressed as U/mg protein [15]. Catalase activity was determined by measuring the decomposition of hydrogen peroxide into water and oxygen, with spectrophotometric analysis at 240 nm, and results expressed as U/mg protein [16]. GSH levels were quantified using Ellman’s reagent method, which measures the reduction of 5,5-dithiobis-(2-nitrobenzoic acid) (DTNB) by GSH, and results were expressed as µmol/mg protein [17].

Statistical analysis

All statistical analyses were performed using InStat (GraphPad, San Diego, CA, USA). Data were analyzed using one-way ANOVA and Tukey’s multiple comparison test for post-hoc analysis. Results were expressed as mean ± standard deviation (SD), and a p-value of <0.05 was considered statistically significant.

## Results

Evaluation of antioxidant supplementation in chelation therapy for lead-induced oxidative stress

The effectiveness of antioxidant supplementation in enhancing chelation therapy for mitigating lead-induced OS was assessed by measuring key OS markers: MDA levels, SOD activity, catalase activity, and GSH levels across all experimental groups: control, lead-exposed, chelation therapy, and combination therapy. These biomarkers provide insights into the degree of oxidative damage and the comparative efficacy of different treatment strategies (Table 1).

**Table 1:** OS markers across different treatment groups All values are expressed in the mean ± SD. * Significant difference with the control group, * ≤ 0.05, ** ≤ 0.01, ***≤ 0.001 SD: standard deviation, MDA: malondialdehyde, SOD: superoxide dismutase, GSH: glutathione, CAT: catalase, p: probability value, F: statistical factor, OS: oxidative stress

	Control group	Lead-exposed group	Chelation therapy group	Combination therapy group	F	P
MDA	1.2 ± 0.36	5.8 ± 0.49***	3.4 ± 0.46***	2.1 ± 0.46*	124.75	0.0001
CAT	8.1 ± 0.3	3.4 ± 0.1***	6.2 ± 0.4***	7.7 ± 0.3***	241.54	0.0001
GSH	9 ± 0.4	2.5 ± 0.2***	6.8 ± 0.5***	8.3 ± 0.4*	307.42	0.0001
SOD	7.8 ± 0.5	3.2 ± 0.4***	5.51 ± 0.2***	6.93 ± 0.3*	126.04	0.0001

The results indicate that the lead-exposed group exhibits the highest OS, as evidenced by significantly elevated MDA (5.8 ± 0.49) and reduced antioxidant enzyme levels (CAT, GSH, and SOD). The control group maintains the lowest MDA (1.2 ± 0.36) and the highest antioxidant levels, serving as the baseline for comparison. The combination therapy and chelation therapy groups demonstrate partial restoration of antioxidant defenses, with the combination therapy group showing near-normal GSH (8.3 ± 0.4) and SOD (6.93 ± 0.3), suggesting a protective effect against oxidative damage. The statistical significance (p < 0.0001) confirms these differences, highlighting the impact of treatments on OS modulation.

MDA Levels (Lipid Peroxidation Marker)

MDA levels, a key indicator of lipid peroxidation and OS, were significantly elevated in the lead-exposed group (5.8 ± 0.49 nmol/mg protein), reflecting severe oxidative damage due to lead toxicity. Chelation therapy reduced MDA levels to 3.4 ± 0.46 nmol/mg protein, indicating a partial reduction in OS. However, combination therapy further decreased MDA levels to 2.1 ± 0.46 nmol/mg protein, bringing them closer to control levels (1.2 ± 0.36 nmol/mg protein). The substantial reduction in MDA levels with combination therapy underscores its superior efficacy in mitigating lipid peroxidation and oxidative damage compared to chelation therapy alone (Table 1).

SOD Activity (Antioxidant Enzyme)

SOD activity, a crucial antioxidant defense mechanism against superoxide radicals, was significantly reduced in the lead-exposed group (3.2 ± 0.4 U/mg protein), indicating compromised cellular antioxidant capacity. Chelation therapy improved SOD activity to 5.51 ± 0.2 U/mg protein, demonstrating partial restoration. Notably, combination therapy further enhanced SOD activity to 6.93 ± 0.3 U/mg protein, approaching the control group's level (7.8 ± 0.5 U/mg protein). This finding highlights the critical role of antioxidants in restoring antioxidant defenses and reducing OS more effectively than chelation therapy alone (Table 1).

Catalase Activity (Antioxidant Enzyme)

Catalase activity, which protects cells from hydrogen peroxide-induced oxidative damage, was significantly diminished in the lead-exposed group (3.4 ± 0.1 U/mg protein). Chelation therapy partially restored catalase activity to 6.2 ± 0.4 U/mg protein, while combination therapy almost normalized catalase activity to 7.7 ± 0.3 U/mg protein, closely aligning with the control group (8.1 ± 0.3 U/mg protein). These results suggest that antioxidants effectively enhance enzymatic defense mechanisms against OS, with combination therapy providing superior protection compared to chelation alone (Table 1).

GSH Levels (Major Antioxidant)

GSH, a vital intracellular antioxidant, was significantly depleted in the lead-exposed group (2.5 ± 0.2 µmol/mg protein), reflecting severe OS. Chelation therapy improved GSH levels to 6.8 ± 0.5 µmol/mg protein, indicating partial recovery. However, combination therapy restored GSH levels to 8.3 ± 0.4 µmol/mg protein, nearing the control group’s level (9.0 ± 0.4 µmol/mg protein). This significant improvement demonstrates that adding antioxidants to chelation therapy enhances GSH replenishment, offering superior protection against oxidative damage compared to chelation therapy alone (Table 1).

ANOVA analysis

A one-way ANOVA was performed to statistically validate the differences in OS markers across the treatment groups (Table 1).

Interpretation

The ANOVA revealed the F-value of 2329, with a specific F-value of 57 for MDA levels, indicating a significant difference between the treatment groups' means and the variation within the groups. The p-value was calculated as 2.47 × 10⁻²¹, far below the 0.05 threshold, confirming that the differences in MDA levels among the groups are statistically significant. These findings suggest that the treatments substantially impacted OS markers, particularly in the combination therapy group, where MDA levels were notably lower (Table 1).

## Discussion

The present study demonstrates the effects of combining chelation therapy with antioxidant supplementation (vitamin C) in mitigating lead-induced OS in rats. Compared to chelation therapy alone, the significant improvements in OS markers in the combination therapy group underscore the critical role of antioxidants in neutralizing ROS and enhancing the efficacy of chelation therapy.

One of the most striking outcomes of this study is the significant reduction in MDA levels, a key marker of lipid peroxidation, in the combination therapy group. MDA levels in the chelation therapy group were lower than in the lead-exposed group, but the combination therapy group exhibited MDA levels nearly approaching those of the control group. This indicates that chelation therapy alone cannot fully reverse lipid peroxidation, but when combined with antioxidants, there is a marked reduction in oxidative damage to cell membranes. These results confirm earlier findings that antioxidant supplementation can reduce lipid peroxidation in lead-exposed models [9,18]. The ability of antioxidants to reduce lipid peroxidation may be attributed to their capacity to scavenge free radicals and prevent the chain reactions that lead to cellular damage [19].

The study highlights the restoration of antioxidant enzyme activities, including SOD and catalase, in the combination therapy group. Lead exposure significantly impaired these enzymes, reflecting a compromised antioxidant defense system. While chelation therapy alone improved the activities of SOD and catalase, the combination of chelation and antioxidants produced a near-complete restoration of enzyme activity to control levels. This suggests that antioxidants neutralize ROS and restore the endogenous antioxidant defense mechanisms, critical in mitigating OS [20]. Previous studies have demonstrated similar results, where antioxidants like vitamins C and E enhanced the activity of antioxidant enzymes in lead-exposed models [15].

The elevated levels of GSH, a major intracellular antioxidant, in the combination therapy group further support the enhanced efficacy of this treatment. GSH plays an important role in detoxifying ROS and maintaining cellular redox balance. Lead exposure resulted in significant depletion of GSH, indicative of heightened OS and reduced cellular capacity to combat free radicals. Although chelation therapy improved GSH levels, the combination therapy effectively restored GSH to near-normal levels. This finding is consistent with earlier reports suggesting that antioxidant supplementation can enhance GSH levels and, consequently, the overall antioxidant defense system in lead-exposed subjects [16,17].

From a clinical perspective, the results of this study hold significant implications for improving the treatment of lead poisoning. While effective in reducing blood lead levels, chelation therapy falls short of fully addressing the oxidative damage caused by lead exposure. The addition of antioxidants fills this therapeutic gap, offering a more holistic approach to managing lead toxicity. Antioxidant supplementation could potentially reduce the long-term complications of lead poisoning, which are often associated with chronic OS and cellular damage. This aligns with growing evidence that suggests a multi-faceted treatment strategy combining chelation agents with antioxidants provides better clinical outcomes than chelation alone [21].

Moreover, the study's findings open the door for further investigation into using other antioxidants or combinations with chelating agents. While vitamins C and E were chosen due to their well-established roles in reducing OS, other natural antioxidants, such as flavonoids or plant-derived compounds, may offer additional benefits. In subsequent research, it may be possible to investigate the utilization of these chemicals in conjunction with chelation therapy to determine the most efficient treatment regimens for lead poisoning treatments. In addition, more studies should concentrate on the long-term benefits of antioxidant supplementation in clinical settings, particularly in populations exposed to lead for an extended period or those at a high risk of lead toxicity. Understanding the long-term benefits of combination therapy in reducing OS-related complications could be instrumental in improving treatment protocols for affected populations [9,12].

Another important aspect that warrants further investigation is the dosage and duration of antioxidant supplementation. While this study used specific doses of vitamins C and E, optimizing the dosage based on factors such as the severity of lead exposure, the patient's age, and an overall health condition could lead to more tailored treatment plans. Additionally, the duration of antioxidant therapy post-chelation remains an open question. It is plausible that extending antioxidant supplementation beyond the duration of chelation therapy could provide continued protection against residual OS, particularly in cases where lead remains stored in bone and tissue for extended periods [22].

Furthermore, the mechanisms by which antioxidants interact with chelating agents to enhance therapeutic outcomes deserve closer scrutiny. Although antioxidants have been shown to reduce OS, their specific interactions with lead and chelating agents, such as EDTA, are not fully understood. For instance, antioxidants might influence the kinetics of chelation, potentially enhancing the rate at which lead is mobilized and excreted. This interaction could be critical in determining the overall efficacy of combination therapy and minimizing chelation's side effects.

While this study utilized a rat model in translational research, the findings provide a compelling basis for clinical trials in human populations. Rats are physiologically similar to humans in many respects, but variations in metabolism, immune response, and antioxidant capacity could influence the outcomes of similar treatments in humans. Clinical trials would help verify whether the benefits observed in rats, such as improved OS markers and restored antioxidant enzyme activities, can be replicated in humans with lead poisoning. Moreover, such trials could explore integrating antioxidant supplementation into routine clinical practice for lead detoxification [11,21].

It is also worth considering the broader public health implications of these findings. Lead poisoning continues to be a significant global health issue, particularly in industrialized regions and low-income communities where exposure to environmental lead is prevalent. Adding antioxidants to chelation therapy could potentially reduce the burden of lead toxicity, improving outcomes for large populations affected by this environmental hazard. Public health initiatives that focus on early intervention, dietary supplementation with antioxidants, and community education could be crucial in mitigating the impact of lead poisoning, especially among vulnerable populations such as children and pregnant women [20].

Limitations and future research

It is important to admit that the current study has a few limitations. First, due to the physiological differences between different species, it is possible that the findings of this study will not be completely applicable to human beings. Second, the research was only conducted on one vitamin C antioxidant. Other antioxidants, such as those naturally present in the human diet, might have additional protective effects against lead-induced OS. Future research should explore the synergistic effects of different antioxidants and their combinations to optimize treatment strategies. Additionally, the long-term effects of combined chelation and antioxidant therapy on lead-induced neurotoxicity, nephrotoxicity, and reproductive toxicity, among other health outcomes, should be examined to provide a more comprehensive understanding of the treatment’s benefits [17].

Nevertheless, this kind of study offers some useful results; however, it is crucial to note that this work was performed on rats, and additional research is necessary to reveal similar results in people. Further, the research involved only one antioxidant, vitamin C. Hence, future research could involve other antioxidants, higher doses, and other treatment durations. Exploring the chronic effects of combined therapy in the clinic will be important in applying the current results to realistic clinical management.

## Conclusions

It is evident from our study that antioxidant supplementation has the potential to improve the efficiency of chelation therapy in lead-induced OS in a rat model. The levels of OS markers were reduced by combination therapy compared to chelation therapy only, and the results indicate that a multiple-drug strategy is a better management plan for lead poisoning. These findings suggest clinical significance for reducing OS to enhance the clinical management of lead toxicity.
